# *Actinomyces naeslundii*: An Uncommon Cause of Endocarditis

**DOI:** 10.1155/2015/602462

**Published:** 2015-11-30

**Authors:** Christopher D. Cortes, Carl Urban, Glenn Turett

**Affiliations:** ^1^The Dr. James J. Rahal, Jr. Division of Infectious Diseases, NewYork-Presbyterian Queens, Flushing, NY 11355, USA; ^2^Department of Medicine, Weill Cornell Medical College, New York, NY 10065, USA

## Abstract

*Actinomyces* rarely causes endocarditis with 25 well-described cases reported in the literature in the past 75 years. We present a case of prosthetic valve endocarditis (PVE) caused by* Actinomyces naeslundii*. To our knowledge, this is the first report in the literature of endocarditis due to this organism and the second report of PVE caused by* Actinomyces*.

## 1. Introduction

*Actinomyces naeslundii* is a Gram positive anaerobic bacillary commensal of the human oral cavity. Septicemia with this organism is uncommon and poses an increased risk of subacute and chronic granulomatous inflammation, which can affect all organ systems via hematogenous spread [1]. We discuss a rare case of endocarditis caused by this organism.

## 2. Case Presentation

A 51-year-old Hispanic woman, with a history of hypothyroidism, asthma, and aortic stenosis requiring a bioprosthetic aortic valve replacement, presented with fever, nausea, and a 23-pound weight loss over the prior two months. The valve was excised at another hospital prior to patient's presentation to us and no pathology information was available. She also complained of diffuse myalgias, erythematous lesions over the arms and legs, and clouded vision. One month prior to aortic valve replacement, she had dental implants inserted. Her symptoms began two months after valve replacement surgery. Physical examination on presentation was notable for a febrile (*T*_MAX_ = 103.1 F) woman in mild distress, but hemodynamically stable. Pertinent findings included poor dentition with apparent tooth decay and fracture below the gum line of two adjacent teeth, small punctate lesions over her upper and lower extremities, Roth spots on ophthalmoscopic exam, a well-healed sternotomy scar, and a grade III/VI holosystolic decrescendo murmur. Abnormal laboratory tests included a WBC count of 16.5 K/*μ*L (reference range: 4.8–10.8 K/*μ*L) with 88.5% neutrophils and C-reactive protein (CRP) 7.02 mg/L (reference range: 0.03–0.49 mg/dL). Only one set of blood cultures was drawn prior to empirically starting vancomycin and ceftriaxone (the patient had a vague history of a nonlife threatening penicillin allergy). The aerobic bottle showed bacterial growth at 26.3 hours and in the anaerobic bottle at 57.1 hours. They were subsequently inoculated on anaerobic blood agar and Brucella Laked Blood Agar with Kanamycin and Vancomycin (LKV). The isolate was identified as* Actinomyces naeslundii* by VITEK 2 ANC ID Card (BioMerieux) anaerobic and* corynebacterium* identification. Vancomycin was discontinued and ceftriaxone alone was used.

A transesophageal echocardiogram demonstrated trace mitral and tricuspid valve regurgitation, but no definitive vegetation. Repeat blood cultures drawn on day two of admission showed no growth and she defervesced within two days. After 7 days of treatment, her leukocytosis resolved and she was no longer symptomatic. No posttreatment echo was done seeing pretreatment and 10 days into treatment, studies showed no aortic valve dehiscence or regurgitation.

A CRP repeated after 21 days was 0.8 mg/L. Four weeks into therapy, ceftriaxone was changed to ertapenem due to a febrile reaction attributed to ceftriaxone. The patient received a total of sixteen weeks of appropriate intravenous antibiotic therapy. She was evaluated by an allergy/immunology specialist and was cleared to receive amoxicillin which then was started at a dose of 1 gram every 12 hours for a planned twelve-month total course of antibiotics. The patient also had her tooth extracted after discharge from the hospital and she follows up every six months with her dentist. Eight weeks later, she presented to the clinic for follow-up. She was well compliant with and tolerating amoxicillin. Surveillance blood cultures drawn then were sterile. Two weeks after completion of the twelve months of antibiotics, she remained well and surveillance cultures drawn on that visit remained negative.

## 3. Discussion

We present an unusual case of* Actinomyces naeslundii* endocarditis in an immunocompetent individual. According to the modified Duke's criteria, our patient had 4 minor criteria classifying her condition as possible endocarditis. The likelihood of endocarditis was strengthened by recent dental work followed by aortic valve replacement surgery and ongoing poor dentation; persistent fevers two months after surgery; bacteremia with* Actinomyces naeslundii*, which is associated with early caries and tooth decay [2]; significant weight loss; evidence of immunologic sequelae (Roth spots) and an elevated inflammatory marker (CRP). Respecting the possibility of prosthetic valve endocarditis prompted aggressive treatment with an extended course of parenteral followed by oral antibiotics.

*Actinomyces* bacteremia is well described in the dental literature following dental procedures [3]. However,* Actinomyces* species rarely cause endocarditis. Actinomycosis is more commonly associated with cervicofacial, pelvic, and thoracic deep-seated infections. There is no specific recommended duration of therapy for* Actinomyces* endocarditis. For patients with deep-seated thoracic infections, 2 to 6 weeks of intravenous antibiotics are advised, followed by oral penicillins for six to twelve months [4].

An extensive review of the literature since 1939 revealed 24 well-described cases of endocarditis due to* Actinomyces* including six cases of* Actinomyces* species [5–9], two of* Actinomyces bovis* [10, 11], four of* Actinomyces viscosus* [12–15], two cases of* Actinomyces meyeri* [16, 17], three of* Actinomyces israelii* [18–20], two of* Actinomyces neuii* [21, 22], and one of each of* Actinomyces septicus* [23],* Actinomyces muris* [24],* Actinomyces pyogenes* [25],* Actinomyces funkei* [26], and* Actinomyces odontolyticus* [27] (Table 1). Almost half of these patients were noted to have predisposing cardiac pathology. One recent report described a case of eustachian valve endocarditis caused by* Actinomyces turicensis* [28]. Four of the 20 patients who received therapy died from pulmonary edema and acute respiratory failure. Three patients underwent aortic valve replacement surgery and survived. Most of the patients received a beta-lactam antibiotic (mainly penicillin), but few received other classes of antibiotics (glycopeptide, sulfonamide, aminoglycoside, and chloramphenicol). In 15 patients, duration of antibiotic therapy was stated and ranged from 24 days to 13 months (mean 21.4 weeks, median 16 weeks).

To our knowledge, this is the first case of* Actinomyces naeslundii* endocarditis reported in the literature to date and the second of prosthetic valve endocarditis caused by* Actinomyces* [22]. Grundmann et al. described a case of* Actinomyces neuii* prosthetic valve endocarditis also managed successfully with medical treatment alone (6-week intravenous regimen of penicillin followed by oral amoxicillin for 11 months) [22].

Similar to the treatment of other serious* Actinomyces* infections, endocarditis requires several weeks of intravenous antibiotic therapy, followed by six to twelve months of oral therapy. High doses of penicillin, for a prolonged period of time, remain the therapy of choice [29].

## Figures and Tables

**Table 1:** Case reports of endocarditis caused by *Actinomyces* species.

Reference	Age/sex	Organism	Valve	Surgery	Therapy	Outcome
Uhr, 1939 [10]	24 M	*Actinomyces bovis*	Aortic/Mitral	No	Sulfathiazole	Dead
Beamer et al., 1945 [5]	55 M	*Actinomyces *sp.	Aortic/Mitral	No	None	Dead
Blevins and Mac Neal, 1946 [23]	UK	*Actinomyces septicus*	Mitral	No	None	UK
MacNeal et al., 1946 [6]	39 M	*Actinomyces *sp.	Mitral	No	PCN	Alive
Wedding, 1947 [7]	37 M	*Actinomyces *sp.	Mitral	No	Sulfathiazole	Dead
Wedding, 1947 [7]	71 F	*Actinomyces *sp.	Aortic	No	None	Dead
Stokes et al., 1951 [24]	27 F	*Actinomyces muris*	Mitral	No	Chloramphenicol	Alive
Walters et al., 1962 [11]	43 F	*Actinomyces bovis*	Aortic/Mitral	No	PCN	Alive
Dutton and Inclan, 1968 [18]	6 M	*Actinomyces israelii*	Mitral	No	PCN	Dead
Gutschik, 1976 [12]	70 M	*Actinomyces viscosus*	Mitral	No	PCN	Alive
Lam et al., 1993 [19]	65 M	*Actinomyces israelii*	Aortic/Mitral	No	PCN	Alive
Moffatt et al., 1996 [16]	48 M	*Actinomyces meyeri*	Aortic	Yes, AVR	PCN	Alive
Reddy et al., 1997 [25]	64 M	*Actinomyces pyogenes*	Aortic	No	Multiple	Dead
Hamed, 1998 [13]	81 M	*Actinomyces viscosus*	Aortic	No	Ceftizoxime	Alive
Huang et al., 1998 [17]	55 M	*Actinomyces meyeri*	Mitral	No	Ampicillin-sulbactam	Alive
Mardis and Many Jr., 2001 [14]	38 M	*Actinomyces viscosus*	Mitral	No	Multiple, PCN	Alive
Westling et al., 2002 [26]	40 F	*Actinomyces funkei*	Tricuspid	No	Multiple	Alive
Oh et al., 2005 [27]	33 M	*Actinomyces odontolyticus*	Tricuspid	No	Ceftriaxone (CTX)	Alive
Julian et al., 2005 [15]	43 F	*Actinomyces viscosus*	Aortic	Yes, AVR	Amp, CTX, and vancomycin	Alive
Cohen et al., 2007 [21]	68 M	*Actinomyces neuii*	Aortic	Yes, AVR	Amp, CTX, and gentamicin	Alive
Oddó and Ayala, 2007 [8]	34 M	*Actinomyces *sp.	Mitral	No	UK	Dead
Grundmann et al., 2010 [22]	66 M	*Actinomyces neuii*	Aortic (prosthetic)	No	PCN	Alive
Adalja and Vergis, 2010 [20]	87 M	*Actinomyces israelii*	Mitral	No	PCN	Alive
Mehrzad et al., 2013 [9]	49 M	*Actinomyces *sp.	Tricuspid	No	Multiple	Alive
Kottam et al., 2015 [28]	30 F	*Actinomyces turicensis*	Eustachian	No	Multiple, pen, and CTX	Alive
Present Report	51 F	*Actinomyces naeslundii*	Aortic (prosthetic)	No	Vanco, CTX, ertapenem, and amoxicillin	Alive

UK: unknown, PCN: penicillin, CTX: ceftriaxone, Amp: ampicillin, Gent: gentamicin, Vanc: vancomycin, Ert: ertapenem, and ^*∗*^valve replacement surgery.
